# Advances in mitochondrial compartmentalization for terpenoid synthesis in *Saccharomyces cerevisiae*

**DOI:** 10.3389/fbioe.2026.1910253

**Published:** 2026-08-28

**Authors:** Chuyi Zheng, Tian Ma

**Affiliations:** 1 Department of Biomedical Engineering, Southern University of Science and Technology, Shenzhen, Guangdong, China; 2 Shenzhen University of Advanced Technology, Shenzhen, China; 3 Shenzhen Institutes of Advanced Technology, Chinese Academy of Sciences, Shenzhen, China

**Keywords:** acetyl coenzyme A, compartmentalization, mitochondria, *Saccharomyces cerevisiae*, terpenoids

## Abstract

Mitochondria provide a unique enriched acetyl coenzyme A reservoir and an ideal redox environment, offering an efficient supply for the biosynthesis of terpene precursors and terpene products. This comprehensive review focuses on the engineering of mitochondrial compartmentalization in *Saccharomyces cerevisiae* to enhance terpene production. We systematically outline the applications from hemiterpenes to tetraterpenes and elaborate on how advanced strategies, such as key enzyme mitochondrial targeting strategy, whole-pathway mitochondrial targeting strategy, dual cytoplasmic-mitochondrial compartmentalization strategy, and multi-organelle synergistic compartmentalization strategy, can effectively improve precursor availability while mitigating metabolic interference and cytotoxic effects. In addition, we critically examine the discussions and long-term challenges associated with these methods. Finally, we highlight the transformative potential of mitochondrial compartmentalization to pioneer future innovations in complex terpene synthesis engineering.

## 1 Introduction

Terpenoids are a large class of natural organic molecules derived from isoprene units. According to the number of isoprene skeletons, terpenoids can be divided into hemiterpenes, monoterpenes, sesquiterpenes, diterpenes, triterpenes, and tetraterpenes (Chandran et al., 2011). They have received extensive attention due to their important biological activities and application values in fields such as medicine, food, spices, cosmetics, and energy. They exhibit various biological activities, such as anti-inflammatory, antibacterial, and antitumor effects, in drug development and are often used in the fragrance and natural product markets (Xia et al., 2024). Currently, terpenoids are mainly obtained through plant extraction and chemical synthesis. However, the low plant content, long production cycles, complex chemical synthesis processes, and high environmental costs create obvious bottlenecks for large-scale production (Jin et al., 2022). Therefore, constructing microbial cell factories to synthesize terpenoids in a sustainable, low-cost, and green way has become a research hotspot, especially engineering microbial platforms such as *Saccharomyces cerevisiae* (Wang et al., 2025).

The naturally occurring mevalonate (MVA) pathway in *S. cerevisiae* can convert acetyl coenzyme A (acetyl-CoA) into the terpene precursors isopentenyl pyrophosphate (IPP)/dimethylallyl pyrophosphate (DMAPP) and is the fundamental pathway for the synthesis of various terpenoids (Christianson, 2017). The key enzymes in this pathway include truncated 3-hydroxy-3-methylglutaryl-coenzyme A reductase (tHMG1, the key rate-limiting enzyme of the MVA pathway) (Ro et al., 2006), farnesyl pyrophosphate synthase (ERG20, catalyzing the production of the sesquiterpenoid precursor farnesyl pyrophosphate (FPP)) (Ignea et al., 2014), and isopentenyl pyrophosphate isomerase (catalyzing the reversible conversion between IPP and DMAPP) (Berthelot et al., 2012). Downstream terpene synthases (such as linalool synthase, limonene synthase and farnesene synthase) (Kutyna and Borneman, 2018) convert precursors into specific terpenoid products. However, terpenoid synthesis in the cytoplasm is often restricted by several core factors. Firstly, there is an insufficient supply of key precursors in the MVA pathway; secondly, there is an imbalance of cofactors such as NADPH and ATP; thirdly, intermediate metabolites and products are toxic to cells; fourthly, competition exists between the heterologous pathway and native cellular pathways, ultimately leading to a reduction in the maximum achievable product titer (Chen et al., 2023; Yin et al., 2024). Notably, the cytotoxicity of intermediate metabolites or final products cannot be underestimated. For example, when the concentration of the sesquiterpene derivatives (+)-nootkatone and β-nootkatol in the medium exceeds 100 mg/L, they can significantly inhibit the viability of *S. cerevisiae* cells. The toxicity of β-nootkatol is most likely closely related to its massive accumulation in the yeast endomembrane system, and this accumulation is expected to alter membrane permeability and integrity, as well as impair the function of integral membrane proteins (Gavira et al., 2013). For core terpene intermediate metabolites such as IPP and DMAPP, the cytotoxicity arising from their excessive accumulation in the cytoplasm has become the consensus in the field. At present, the direct molecular mechanism underlying this cytotoxicity in *S. cerevisiae* systems has not been systematically elucidated, but related metabolic engineering studies (Wang et al., 2017; Kim et al., 2021) have indirectly confirmed that the accumulation of these intermediates is a critical bottleneck restricting the flux improvement of the cytoplasmic terpene pathway. Aiming at the aforementioned bottlenecks of terpene synthesis in the cytoplasm, traditional metabolic engineering strategies mainly focus on overexpressing cytoplasmic pathways and weakening competing pathways, which are far from fundamentally solving the problems of metabolic crosstalk and resource allocation.

Subcellular compartmentalization refers to the use of different membranous organelles within the cell (such as mitochondria, peroxisomes, and endoplasmic reticulum) to provide distinct microenvironments for specific metabolic pathways, thereby enhancing precursor supply and alleviating metabolic competition and toxicity (Hammer and Avalos, 2017). Among them, the concentration of acetyl-CoA in mitochondria is 20–30 times that in the cytoplasm, which mainly results from the pyruvate dehydrogenase complex localized in the mitochondrial matrix that can directly catalyze the oxidative decarboxylation of pyruvate to generate acetyl-CoA. In contrast, the cytoplasm lacks this enzyme complex, and pyruvate must be indirectly converted to acetyl-CoA via the multi-step reactions of the pyruvate dehydrogenase bypass. Therefore, mitochondria can provide a more direct and efficient supply of acetyl-CoA precursors for terpene biosynthesis (Weinert et al., 2014). Secondly, the highly selective permeability of the inner mitochondrial membrane forms a physical barrier to cytoplasmic metabolism. In the cytoplasm, the core competition for terpenoid synthesis arises from the sterol pathway. FPP generated by ERG20 is largely diverted to sterol synthesis, resulting in a significant diversion of carbon flux (Paradise et al., 2008). In contrast, the double-membrane structure of mitochondria restricts the leakage of FPP into the cytoplasm. It retains the FPP synthesized in mitochondria within the matrix (Yuan and Ching, 2016), enabling it to be more effectively utilized by exogenous terpene synthases and avoiding flowing into the competing sterol pathway in the cytoplasm (Zhang et al., 2025). At the same time, mitochondria can provide a unique cofactor environment and high redox potential, which can provide sufficient impetus for the MVA pathway and downstream redox enzymes. The unique metabolic characteristics of mitochondria mentioned above are highly consistent with the metabolic requirements of terpene precursor biosynthesis, making it an important candidate organelle for compartmentalized metabolic engineering of terpenoids (Duran et al., 2020). The development of this idea began when Farhi et al. (2011) first targeted plant-derived farnesyl diphosphate synthase and terpene synthases to mitochondria and found that the existing FPP pool in mitochondria could directly provide substrates for the targeted terpene synthases. The titers were 8-fold (valencene) and 20-fold (amorpha-4,11-diene) those expressed in the cytoplasm.

**FIGURE 1:**
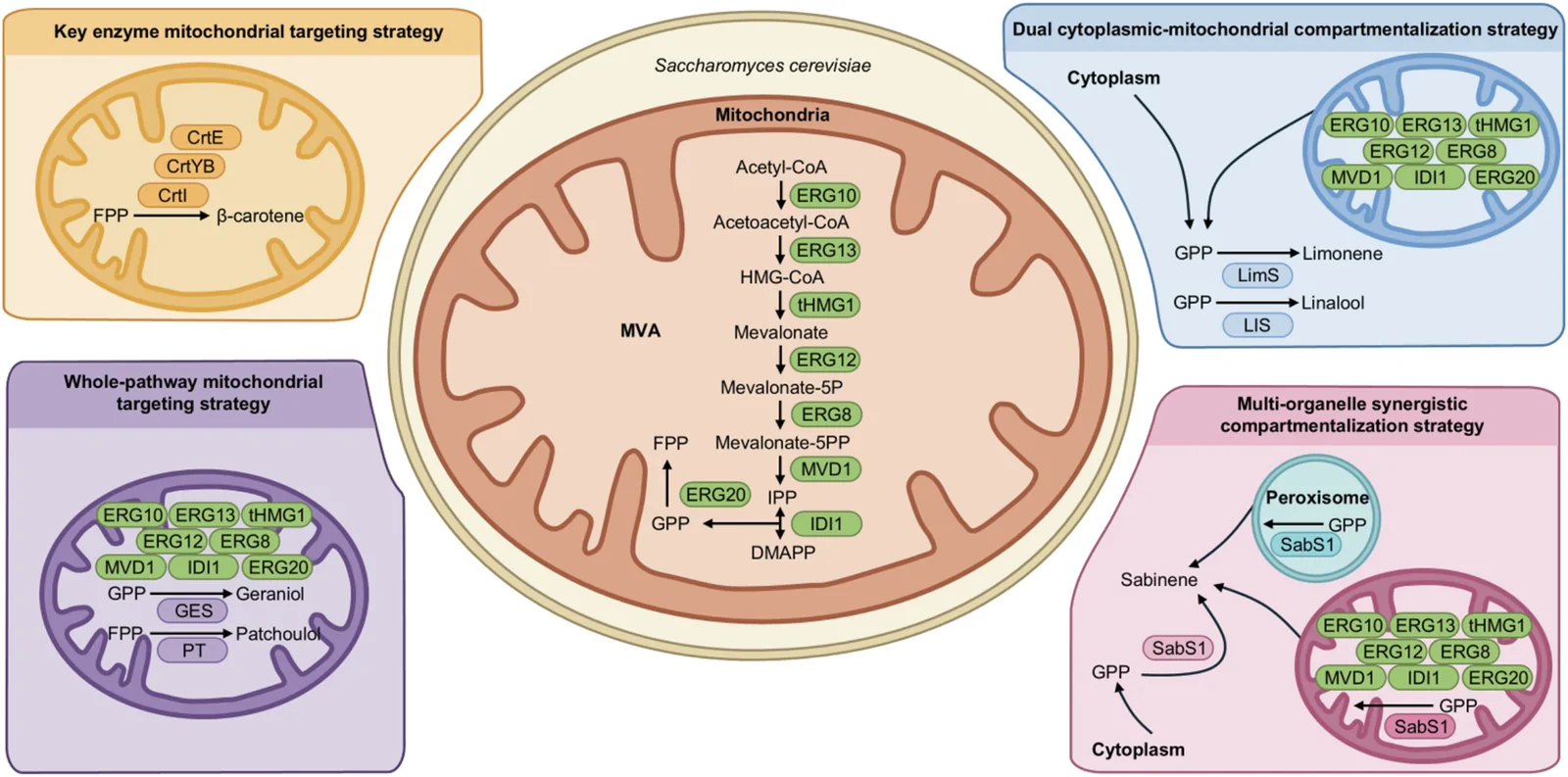
Framework of mitochondrial compartmentalization strategies for the terpenoid synthesis pathway in *Saccharomyces cerevisiae*. Harnessing yeast mitochondria has enabled the production of sabinene, linalool, geraniol, limonene, patchoulol, and β-carotene. The native enzymes in the MVA pathway that are overexpressed are labeled in green, and the heterologous enzymes targeted for expression in the corresponding compartments are labeled in other colors. ERG10, acetyl-CoA C-acetyltransferase; ERG13, 3-hydroxy-3-methylglutaryl-CoA synthase; tHMG1, truncated 3-hydroxy-3-methylglutaryl-CoA reductase; ERG12, mevalonate kinase; ERG8, phosphomevalonate kinase; MVD1, mevalonate pyrophosphate decarboxylase; IDI1, isopentenyl diphosphate isomerase; ERG20, farnesyl pyrophosphate synthetase; CrtE, geranylgeranyl pyrophosphate synthase; CrtYB, bifunctional phytoene synthase/lycopene cyclase; CrtI, phytoene dehydrogenase; SabS1, sabinene synthase; LIS, linalool synthase; GES, geraniol synthase; LimS, limonene synthase; PT, patchoulol synthase; Acetyl-CoA, Acetyl coenzyme A; Acetoacetyl-CoA, Acetoacetyl coenzyme A; HMG-CoA, 3-hydroxy-3-methylglutaryl coenzyme A; Mevalonate-5P, Mevalonate 5-phosphate; Mevalonate-5PP, Mevalonate 5-diphosphate; IPP, isopentenyl pyrophosphate; DMAPP, dimethylallyl pyrophosphate; GPP, geranyl disphosphate; FPP, farnesyl pyrophosphate; MVA, mevalonate.

In light of the above background, this review focuses on the application of mitochondrial compartmentalization in terpenoid synthesis in *S. cerevisiae* and systematically discusses the current research progress and future research directions of mitochondrial compartmentalization, providing a theoretical basis and practical reference for subsequent research in this field (Figure 1).

## 2 Mitochondrial compartmentalization in terpenoid synthesis

The mitochondrial compartmentalization strategy has become an important means to improve the terpenoid production in yeast. In recent years, significant progress has been made in this strategy in two dimensions, expanding the range of product types and iterating on engineering methods (Table 1).

**TABLE 1:** Application cases of mitochondrial compartmentalization in terpenoid biosynthesis in *Saccharomyces cerevisiae*.

References	Strategy type	Product category	Terpenoid products	Culture system and fermentation culture mode	Titers
(Farhi et al., 2011)	C. dual cytoplasmic-mitochondrial compartmentalization strategy	Sesquiterpene	Valencene; amorpha-4,11-diene	Test tubes; two phase partitioning batch culture	Valencene: 1.5 mg/L; amorpha-4,11-diene:∼20 mg/L
(Yuan and Ching, 2016)	B. whole-pathway mitochondrial targeting strategy	Sesquiterpene	Amorpha-4,11-diene	Shake flasks; two-phase batch fermentation	427 mg/L
(Lv et al., 2016)	C. dual cytoplasmic-mitochondrial compartmentalization strategy	Hemiterpene	Isoprene	A 5-L fermenter; fed-batch fermentation	2,527 mg/L
(Yao et al., 2018)	C. dual cytoplasmic-mitochondrial compartmentalization strategy	Hemiterpene	Isoprene	A 5-L fermenter; fed-batch fermentation	11.9 g/L
(Yee et al., 2019)	B. whole-pathway mitochondrial targeting strategy	Monoterpenoid	Geraniol; 8-hydroxygeraniol; nepetalactol	Geraniol and nepetalactol: culture tubes; batch culture 8-hydroxygeraniol: A 2-L vessel; fed-batch fermentation	Geraniol: 43.3 mg/L; 8-hydroxygeraniol: 227 mg/L; nepetalactol: 5.9 mg/L
(Zhang Y. et al., 2020)	C. dual cytoplasmic-mitochondrial compartmentalization strategy	Monoterpenoid	Linalool	A 2-L fermenter; batch fermentation	23.45 mg/L
(Jia et al., 2020)	C. dual cytoplasmic-mitochondrial compartmentalization strategy	Monoterpene	Sabinene	Shake flasks; batch culture	154.9 mg/L
(Dong et al., 2021)	B. whole-pathway mitochondrial targeting strategy	Sesquiterpene	α-Santalene	Shake flasks; two-phase batch fermentation	∼41 mg/L
(Zhu et al., 2021)	C. dual cytoplasmic-mitochondrial compartmentalization strategy	Triterpene	Squalene	A 5-L bioreactor; fed-batch fermentation	21.1 g/L
(Matsumoto et al., 2022)	A. key enzyme mitochondrial targeting strategy	Tetraterpenoid	Carotenoid	Shake flasks; batch culture	0.354 mg/L culture/OD_600_
(Tao et al., 2022)	B. whole-pathway mitochondrial targeting strategy	Sesquiterpenoid	Patchoulol	Shake flasks; batch culture	19.24 mg/L
(Kong et al., 2023)	C. dual cytoplasmic-mitochondrial compartmentalization strategy	Monoterpene	Limonene	A 3-L fermenter; fed-batch fermentation	2.63 g/L
(Yanagibashi et al., 2024)	C. dual cytoplasmic-mitochondrial compartmentalization strategy	Triterpene; Tetraterpene	Squalene; β-carotene	Shake flasks; batch culture	Squalene: 707 nmol/g DCW; β-carotene: 1609 nmol/g DCW
(Ge et al., 2025)	D. multi-organelle synergistic compartmentalization strategy	Diterpene	Miltiradiene	A 5-L bioreactor; fed-batch fermentation	1.02 g/L
(Tang et al., 2025)	D. multi-organelle synergistic compartmentalization strategy	Triterpene	Squalene	A 5-L fermenter; fed-batch fermentation	55.82 g/L
(Wang S. et al., 2026)	C. dual cytoplasmic-mitochondrial compartmentalization strategy	Monoterpenoid	α-Terpineol	A 5-L bioreactor; fed-batch fermentation	81.78 mg/L

Mitochondrial targeting sequences (MTS) are key tools for guiding exogenous enzymes to the mitochondrial matrix. MTS are typically N-terminal amphipathic helical peptide segments. After protein translation, they guide fusion proteins into the mitochondrial matrix through the transport mechanism mediated by the translocase of the outer mitochondrial membrane/translocase of the inner mitochondrial membrane complex and are subsequently cleaved by mitochondrial processing peptidases (Burkhart et al., 2015). Currently, the most widely used MTS is derived from the N-terminal amino acid residues 1–25 of yeast cytochrome c oxidase subunit IV (Cox4) (Hurt et al., 1985), and its effectiveness has been verified in various terpene synthesis studies. Besides, commonly used MTS also include Coq3, Cox6, and Mmf1 (Table 2).

**TABLE 2:** Mitochondrial targeting sequences.

Signal gene name	Source species	Location	Length (AA)	Sequences	References
*COX4*	*S. cerevisiae*	N-terminal	26	MLSLRQSIRF FKPATRTLCS SRYLLQ	(Hurt et al., 1985; Yuan and Ching, 2016; Dong et al., 2021)
*HMI1*	*S. cerevisiae*	C-terminal	36	VTHGYNVQRY NQLRKNFGFY RAYSSLRGCK SVFRRI	(Lee et al., 1999)
*COQ3*	*S. cerevisiae*	N-terminal	35	MLLRSRFLKV IHVRKQLSAC SRFAIQTQTR CKSTD	(Yuan and Ching, 2016; Dong et al., 2021)
*CDC9*	*S. cerevisiae*	N-terminal	53	MRRLLTGCLL SSARPLKSRL PLLMSSSLPS SAGKKPKQAT LARFFTSMKN KPT	(Willer et al., 1999; Dong et al., 2021)
*COX6*	*S. cerevisiae*	N-terminal	41	MLSRAIFRNP VINRTLLRAR PGAYHATRLT KNTFIQSRKY S	(Wright et al., 1984; Dong et al., 2021)
*MMF1*	*S. cerevisiae*	N-terminal	26	MFLRNSVLRT APVLRRGITT LTPVST	(Oxelmark et al., 2000; Dong et al., 2021; Ge et al., 2025)
*HSP10*	*S. cerevisiae*	N-terminal	27	MSTLLKSAKS IVPLMDRVLV QRIKAQA	(Dong et al., 2021)
*SOD2*	*S. cerevisiae*	N-terminal	27	MFAKTAAANL TKKGGLSLLS TTARRTK	(Jensen and Culotta, 2000; Dong et al., 2021)
*HSP60*	*S. cerevisiae*	N-terminal	26	MLRSSVVRSR ATLRPLLRRA YSSHKE	(Dong et al., 2021; Ge et al., 2025)
*SSC1*	*S. cerevisiae*	N-terminal	24	MLAAKNILNR SSLSSSFRIA TRLQ	(Dong et al., 2021)
*KGD2*	*S. cerevisiae*	N-terminal	72	MLSRATRTAA AKSLVKSKVA RNVMAASFVK RHASTSLFKQ ANKVESLGSI YLSGKKISVA ANPFSITSNR FK	(Dong et al., 2021; Mark et al., 2023)
*LAT1*	*S. cerevisiae*	N-terminal	29	MSAFVRVVPR ISRSSVLTRS LRLQLRCYA	(Dong et al., 2021)
*LSC2*	*S. cerevisiae*	N-terminal	39	MYSRKSLSLI SKCGQLSRLN AQAALQARRH LSIHEYRSA	(Dong et al., 2021)
*ALD4*	*S. cerevisiae*	N-terminal	32	MFSRSTLCLK TSASSIGRLQ LRYFSHLPMT VP	(Noree, 2018; Dong et al., 2021)
*ACO1*	*S. cerevisiae*	N-terminal	24	MLSARSAIKR PIVRGLATVS NLTR	(Regev-Rudzki et al., 2008; Dong et al., 2021)
*CIT1*	*S. cerevisiae*	N-terminal	38	MSAILSTTSK SFLSRGSTRQ CQNMQKALFA LLNARHYS	(Dong et al., 2021)
*LPD1*	*S. cerevisiae*	N-terminal	29	MLRIRSLLNN KRAFSSTVRT LTINKSHDV	(Dong et al., 2021; Ge et al., 2025)
*ILV5*	*S. cerevisiae*	N-terminal	48	MLRTQAARLI CNSRVITAKR TFALATRAAA YSRPAARFVK PMITTRGL	(Dong et al., 2021)
*ADH3*	*S. cerevisiae*	N-terminal	28	MLRTSTLFTR RVQPSLFSRN ILRLQSTA	(Dong et al., 2021)
*IDH2*	*S. cerevisiae*	N-terminal	27	MLRNTFFRNT SRRFLATVKQ PSIGRYT	(Dong et al., 2021)
*MDH1*	*S. cerevisiae*	N-terminal	18	MLSRVAKRAF SSTVANPY	(Thompson and McAlister-Henn, 1989; Dong et al., 2021)

The underlined sequences represent the shortened sequences used in the references.

### 2.1 Expansion of product synthesis from hemiterpenes to tetraterpenes

The mitochondrial compartmentalization strategy was initially applied to the synthesis of sesquiterpenes (Farhi et al., 2011) and subsequently extended to hemiterpenes, monoterpenes, diterpenes, triterpenes, and even tetraterpenes, demonstrating its versatility in producing various terpene skeletons.

In the synthesis of hemiterpene, Lv et al. (2016) targeted the MVA pathway (containing 7 genes) to the mitochondria of *S. cerevisiae* via MTS, and combined with cytoplasmic engineering modifications, including overexpression of *tHMG1* and downregulation of *ERG20* expression, the strain finally achieved efficient isoprene biosynthesis.

In the synthesis of monoterpene, Zhang Y. et al. (2020) reconstructed the MVA pathway in both the mitochondria and the cytoplasm, and targeted the linalool synthase fused with the mutant ERG20^F96W−N127W^ to the mitochondria. Combined with weakening endogenous *ERG20* and adding mevalonolactone, the linalool titer reached 23.45 mg/L, about 19-fold that of the initial strain (1.24 mg/L).

In the synthesis of sesquiterpene, Yuan and Ching (2016) targeted amorpha-4,11-diene synthase to mitochondria by fusing it with the Coq3 mitochondrial targeting signal, which increased the product titer by 63% relative to cytoplasmic expression. Subsequently, the team fused each of the eight enzymes of the FPP synthesis pathway to the Cox4 mitochondrial targeting signal, and integrated the corresponding expression cassettes into the yeast chromosome, thereby achieving functional compartmentalized expression of the entire pathway in mitochondria. The final engineered strain produced amorpha-4,11-diene at a titer of 427 mg/L.

The breakthrough in diterpene production came from the research conducted by Ge et al. (2025). Aiming at the bottlenecks where the endogenous MVA pathway in *S. cerevisiae* is strictly regulated, with insufficient precursor supply and high cofactor demand, this work combined the orthogonal isopentenol utilization pathway (IUP) with a multi-organelle synergistic compartmentalization strategy, and realized the assembly-line catalysis from IPP/DMAPP to miltiradiene in yeast for the first time. Taking advantage of the orthogonality between IUP and endogenous metabolism, as well as its unique feature of requiring ATP as the primary cofactor, the researchers introduced IUP into mitochondria to fully leverage the abundant ATP pool in this organelle. Meanwhile, peroxisomes were recruited to collaboratively utilize a large amount of acetyl-CoA generated from β-oxidation in this organelle. The upstream enzymes of the MVA pathway, ERG10, ERG13, and tHMG1, were additionally introduced into peroxisomes, while the miltiradiene synthesis module was expressed in both the cytoplasm and peroxisomes. This design achieved optimized allocation of precursors and energy across three compartments: mitochondria, peroxisomes, and cytosol. Furthermore, unlike previous multi-enzyme colocalization strategies, this study drew on the oligomerization structure of the natural multifunctional enzyme PaFS as a design inspiration. By screening prenyltransferase domains from multiple sources, it was verified that the fusion of PvPT and tSmCPS-tSmKSL yielded the highest miltiradiene production. On this basis, the artificial multifunctional enzyme tSmCPS-tSmKSL-PvPT was constructed, realizing the assembly-line catalysis from IPP/DMAPP to miltiradiene. The shake-flask titer of miltiradiene was increased to 146.1 mg/L via compartment combinatorial engineering, and further optimized to 414.4 mg/L through artificial multifunctional enzyme engineering. Finally, the titer reached 1.02 g/L in a 5 L bioreactor through fed-batch fermentation, which was 60.8 times higher than that of the initial strain. This research provides a systematic path from pathway design to titer improvement for the synthesis of complex diterpenoids.

The large-scale production of the triterpene squalene was accomplished by Zhu et al. (2021). By targeting the MVA pathway to the mitochondria, overexpressing tHMG1 in the cytoplasm, and combining with two-stage fed-batch fermentation, the squalene titer reached 21.1 g/L in a 5-L bioreactor, which was the highest record at that time. This study first revealed the toxicity of phosphorylated intermediates resulting from mitochondrial compartmentalization and alleviated it by enhancing intermediate conversion and diverting intermediates to dual pathways.

In the synthesis of tetraterpene, Matsumoto et al. (2022) provide a representative case. They targeted only three carotenoid synthases to mitochondria via MTS. After 48 h of cultivation in medium containing 120 g/L glucose, the product titer reached 0.354 mg/L culture/OD_600_, which was approximately 15.4-fold higher than that of the cytosolic-expression control strain. This study provided preliminary evidence for the feasibility of the mitochondrial compartmentalization strategy for tetraterpene synthesis. Nevertheless, this titer remains far below industrial requirements, indicating substantial room for further improvement.

### 2.2 Iteration of mitochondrial compartmentalization strategies

With further in-depth research, mitochondrial compartmentalization strategies in *S. cerevisiae* have undergone continuous iterations, from the key enzyme mitochondrial targeting strategy to the whole-pathway mitochondrial targeting strategy and dual cytoplasmic-mitochondrial compartmentalization strategy, and then to the multi-organelle synergistic compartmentalization strategy.

Compartmentalized targeting of key enzymes refers to the directional transport of essential pathway enzymes for target product synthesis into mitochondria, thereby exploiting the intrinsic advantages of this organelle to enhance product titers. As noted above, the study by Matsumoto et al. demonstrated that mitochondrial compartmentalization of pathway enzymes can serve as a novel strategy to improve carotenoid production in *S. cerevisiae*. This approach is operationally simple and highly efficient; however, the flux through the upstream pathway ultimately constrains its overall effectiveness. This strategy is simple and efficient, but the flux through the upstream pathway limits its effectiveness.

To further improve precursor supply, researchers developed a whole-pathway mitochondrial targeting strategy, targeting all genes of the complete MVA pathway to the mitochondria of *S. cerevisiae*. For instance, Tao et al. (2022) targeted the MVA pathway enzymes and patchoulol synthase to the mitochondria, resulting in a patchoulol titer of 1.79 mg/L, which was 2.71-fold that of the cytoplasmic expression control strain (0.66 mg/L). Further, by fusing FPP synthase and patchoulol synthase and overexpressing it on a high-copy plasmid, the titer was significantly increased to 19.24 mg/L. The results showed that reconstructing the pathway within the mitochondria allowed the conversion from substrate to product to be completed in a single compartment, maximizing the utilization of the mitochondrial acetyl-CoA pool and thus significantly enhancing the pathway flux. However, excessive exogenous proteins may impose stress on the mitochondria.

The dual cytoplasmic-mitochondrial compartmentalization strategy refers to an engineering method that balances cell growth and product synthesis. It involves either enhancing the natural cytoplasmic terpene synthesis pathway or reconstructing the synthesis pathway in the cytoplasm, while simultaneously targeting and expressing key enzymes in the mitochondria. Yao et al. (2018) obtained the isoprene synthase mutant via saturation mutagenesis, increasing isoprene production by about 4-fold. Subsequently, additional copies of the *MVD1* gene and the *IDI1* gene were introduced into the cytoplasmic and mitochondrial engineered strains to reconstruct the metabolic balance at the IPP/DMAPP node. Finally, the optimized diploid strain produced 11.9 g/L of isoprene in fed-batch fermentation, which was the highest reported level in eukaryotic cells at that time. Kong et al. (2023) dynamically inhibited ERG20, enhanced the supply of acetyl-CoA and NADPH, and combined the dual regulation of cytoplasmic and mitochondrial engineering to increase the limonene titer to 1586 mg/L, which was about 34-fold higher than that of the initial strain (46.96 mg/L). Finally, the limonene titer of 2.63 g/L was achieved in a 3-L bioreactor. The dual cytoplasmic-mitochondrial compartmentalization strategy utilizes the precursor resources in both the cytoplasm and mitochondria, avoiding the flux limitation of a single compartment.

To further expand the scope of compartment utilization, the multi-compartment synergy strategy takes mitochondria as the core synthetic compartment. It combines with other organelles to optimize precursor supply, thereby extending the upgrading of the mitochondrial compartmentalization route. Tang et al. (2025) further synergistically engineered multiple subcellular compartments, such as the endoplasmic reticulum, lipid droplets, and cell wall, based on the high-titer squalene strain constructed by the dual cytoplasmic-mitochondrial compartmentalization strategy. By knocking out the gene encoding citrate synthase to reduce the consumption of acetyl-CoA in peroxisomes and optimizing the supply of NADPH, the final constructed strain achieved a squalene production of 55.82 g/L in a 5-L fermenter, obtaining the highest reported value for squalene synthesis in *S. cerevisiae* to date, demonstrating the effectiveness of the multi-compartment synergistic engineering strategy in increasing the production of terpene compounds.

In summary, the mitochondrial compartmentalization strategy has successfully achieved high production of various terpene compounds from hemiterpenes to tetraterpenes and has iterated from the key enzyme targeting strategy to full mitochondrial reconstitution of the biosynthetic pathway and multi-organelle synergistic compartmentalization strategy, continuously breaking the upper limit of terpene production and fully demonstrating its versatility and high efficiency in the *S. cerevisiae* cell factory.

### 2.3 MTS selection and targeting efficiency

Although the MTS derived from Cox4 is the most widely used, it is not the optimal choice for all scenarios. There are significant differences in the fusion effects of different MTS with various enzymes. For multi-gene co-localization, combinatorial optimization of MTS is a key variable for improving biosynthetic flux. For example, Dong et al. systematically screened six MTS for the co-localization of the α-santalene synthesis pathway. The results showed that there were significant differences in the targeting efficiency of different MTS, and the optimal combination increased α-santalene production to 3.7-fold than that of the control strain (Dong et al., 2021). This study suggests that for the whole-pathway mitochondrial targeting strategy involving multi-gene colocalization, the combined optimization of MTS may bring about considerable titer improvement. It is worth noting that the study by Dong et al. is currently the only original study that systematically screened MTS for terpene synthesis. Therefore, there is still a lack of systematic comparative studies on the targeting efficiency after the fusion of MTS with different enzymes, such as the rules of the targeting efficiency of different MTS for the same enzyme, the compatibility rules of the same MTS for different enzymes, and the influence on protein folding and activity after the fusion of MTS and enzymes. Therefore, in the whole-pathway mitochondrial compartmentalization strategy, the screening and combined optimization of MTS should be considered as an equally important design link as the regulation of enzyme expression levels.

## 3 Application of mitochondrial compartmentalization in terpenoid synthesis

### 3.1 Comparison of compartmentalization in different organelles of *S*. *cerevisiae*

Currently, in the research on the compartmentalization application of terpenoid synthesis in *S. cerevisiae*, according to the differences in target organelle selection and metabolic organization strategies, two mainstream compartmentalization technology schools have emerged. They show different application effects and technical limitations under different product types and fermentation conditions. Studies advocating “mitochondrial optimization” focus on maximizing mitochondria’s synthetic capacity, with targeting pathway enzymes to mitochondria via MTS as the core implementation approach. In addition, mitochondrial morphology engineering is a critical strategy to optimize mitochondrial terpenoid synthesis capacity from the spatial capacity perspective, and knocking out *MDM32* is a typical application of this approach (Yanagibashi et al., 2024). Mdm32 is an inner mitochondrial membrane protein of *S. cerevisiae*. Under physiological conditions, it exists in the form of a distinct complex, with core functions including maintaining the normal tubular morphology and internal membrane structure of mitochondria, stabilizing mitochondrial DNA nucleoids, and ensuring the intracellular movement and normal mtDNA inheritance. Deletion of this gene will lead to abnormal mitochondrial morphology and the formation of giant spherical mitochondria (Dimmer et al., 2005). Based on this physiological property, knocking out *MDM32* can increase the total mitochondrial volume of yeast to 1.8 times than that of the wild type. In engineered strains equipped with the mitochondria-targeted MVA pathway, this operation can further significantly increase the titer of the target product. However, this strategy has multiple limitations. The toxicity caused by the accumulation of phosphorylated intermediates still persists. Mitochondrial morphology modification itself will trigger growth defects in the strain, and Mdm32 deletion will lead to decreased stability of mitochondrial DNA. These problems significantly limit the industrial potential of this strategy.

On the other hand, the school of “peroxisome compartmentalization” believes that peroxisomes have a detoxification function and their quantity and size can be flexibly regulated (Van Der Klei and Veenhuis, 1997; Dusséaux et al., 2020). In recent years, many studies have confirmed that peroxisomes can be used for the synthesis of some monoterpenes and the storage and synthesis of hydrophobic terpenoids (Liu et al., 2020; Zhang C. et al., 2020; Baker et al., 2025; Wang J. et al., 2026). However, this strategy has some natural bottlenecks. For example, acetyl-CoA in peroxisomes is isolated by the membrane and cannot flow out effectively, which becomes the core limitation of terpenoid synthesis. Therefore, some research (Liu et al., 2026) broke through the bottleneck of acetyl-CoA limitation by adopting the decompartmentalization strategy of peroxisomal acetyl-CoA, and the titer of wilforic acid C was increased by more than 100-fold compared with previous studies. At the same time, problems such as insufficient supply of peroxisome cofactors and the inhibition of its capacity by carbohydrate source regulation further restrict its application effect (Ye et al., 2026).

A comparison of the two major compartmentalization strategies reveals that the mitochondrial approach, with its abundant acetyl-CoA pool and highly active energy-metabolism microenvironment, is better suited for terpenoids requiring substantial precursor supply. This strategy has been successfully applied across the entire terpenoid spectrum-from hemiterpenes to tetraterpenes-with varying degrees of titer improvement, demonstrating strong universality and reproducibility. In fed-batch systems for isoprene, limonene, and squalene, mitochondrial compartmentalization maintains stable scale-up performance, highlighting its industrial potential. However, for complex terpenoids with longer pathways and higher cofactor demands, reliance solely on mitochondria often fails to overcome flux bottlenecks.

In contrast, peroxisomal compartmentalization leverages its inherent advantages in lipid metabolism, detoxification, and hydrophobic product storage. It has yielded multiple high-titer examples for monoterpenes, sesquiterpenes, and squalene, with scale-up potential further supported by peroxisome capacity optimization and cytosol-peroxisome synergy. Nevertheless, successful cases remain largely confined to a few product types. Constraints including acetyl-CoA transmembrane utilization, cofactor supply, and organelle capacity mean that its broader applicability to more complex terpenoids requires further validation.

As target products have expanded from mono- and sesquiterpenes to diterpenes and higher-carbon terpenoids, single-compartment strategies increasingly fall short in meeting the combined demands of precursor supply, cofactor balance, and product storage. Consequently, recent high-titer studies have shifted from single-compartment optimization toward multi-organelle synergistic compartmentalization, which exploits metabolic division of labor across organelles to unlock greater biosynthetic potential. Moreover, most gram-per-liter titers have been achieved by combining compartmentalization with precursor enhancement, cofactor optimization, and fermentation process improvements. This suggests that compartmentalization should be viewed as a core modular component within the spatial optimization of metabolic networks, not as a standalone titer-boosting tactic. In practice, the choice of compartmentalization mode should therefore be guided by target terpenoid complexity, production scale, and engineering cost to maximize overall benefits.

### 3.2 Current research gaps in mitochondrial compartmentalization

For the mitochondrial compartmentalization strategy to truly develop into a universally applicable industrial-scale synthesis technology, it still faces a series of fundamental research gaps that have not been clarified. These issues directly limit the expansion of its product spectrum and the further improvement of production performance. Specifically, firstly, there are technical barriers to the improvement of product complexity. Currently, almost all high-titer cases of mitochondrial compartmentalization are concentrated on monoterpenes and sesquiterpenes, whose skeleton construction only requires terpene synthases. However, for the synthesis of diterpenes and higher-order terpenes, cytochrome P450 oxidases are indispensable, yet their functional expression is often constrained by low local enzyme concentrations, suboptimal catalytic properties, and inefficient electron transfer. Although mitochondria are the primary site of heme biosynthesis and their compact inner membrane architecture favors substrate and enzyme concentration, excessive heterologous protein expression impairs mitochondrial function as the central hub of cellular energy metabolism, thereby compromising cell growth (Liu et al., 2025). To overcome these challenges, feasible strategies include enhancing heme supply, optimizing NADPH regeneration, screening compatible CPRs with optimized P450/CPR ratios, and using scaffold proteins or co-expressing cytochrome b5 to facilitate electron transfer. These combined strategies have been validated in the synthesis of diterpenoid carnosic acid, achieving a final titer of 75.18 mg/L in a 5-L fed-batch fermentation (Wei et al., 2022). However, when applying mitochondrial compartmentalization to the biosynthesis of P450-dependent diterpenoids and higher-carbon terpenoids, how to balance the catalytic demands of heterologous P450 enzymes with maintaining mitochondrial function as the energy-metabolism hub remains poorly understood.

Secondly, there are fundamental obstacles in the interaction of transmembrane metabolic fluxes. The inner mitochondrial membrane forms a strict selective barrier for charged phosphorylated metabolites, and the known transmembrane exchange of metabolites almost entirely depends on specific carrier proteins (Palmieri, 2013). When the complete MVA pathway is reconstructed in the mitochondrial matrix, phosphorylated intermediates such as mevalonate-5-P and mevalonate-5-PP will accumulate within mitochondria because they cannot cross the mitochondrial membrane and be exported. It is speculated that these intermediates can be converted into ATP analogues, which severely inhibit the oxidative respiration function and trigger toxic effects (Zhu et al., 2021). Although IPP and DMAPP can partially exit the mitochondria to alleviate toxicity, this process remains limited by unknown transport mechanisms. At present, it has only been identified that the mitochondrial glycine carrier Hem25p in *S. cerevisiae* can moonlight in mediating the import of cytoplasmic IPP into the mitochondrial matrix for coenzyme Q synthesis (Tai et al., 2023). However, there is still a lack of systematic functional identification and research on whether Hem25p has the DMAPP transport capacity, whether it possesses the function of mediating the reverse export of IPP, and whether there is a dedicated member in the mitochondrial carrier family with high selectivity and high activity for C5 pyrophosphate substrates.

Furthermore, there is a lack of systematic analysis of the trans-compartment regulatory effect and quantitative flux matching mechanism of the mitochondrial NADPH generation system. In *S. cerevisiae*, the inner mitochondrial membrane is impermeable to both NADPH and NADH (Outten and Culotta, 2003). The NADPH in the mitochondrial matrix depends on the catalytic reactions mediated by the NAD^+^/NADH kinase *POS5* and NADP^+^-dependent enzymes. Among them, the mitochondrial NADH kinase Pos5p exhibits significantly higher catalytic efficiency for NADH than for NAD^+^; the aldehyde dehydrogenase Ald4p plays an important role when the NADH kinase function of Pos5p is restricted (Miyagi et al., 2009). Key steps in the MVA pathway, such as HMG-CoA reductase, are highly dependent on NADPH, and 2 molecules of NADPH are consumed for each molecule of mevalonate produced (Paramasivan and Mutturi, 2017). In the cytoplasmic terpenoid synthesis of *S. cerevisiae*, the overexpression of tHMG1 is limited by the NADPH cofactor, while overexpressing the mitochondria-targeted NADH kinase Pos5p can significantly improve the squalene titer; in the mitochondrial compartmentalized synthesis pathway, the co-overexpression of *POS5* and *IDP1* can synergistically increase the accumulation of 3-hydroxypropionate, a mitochondrial-localized acetyl-CoA derivative (Paramasivan and Mutturi, 2017; Zhang et al., 2023). The above cases suggest that enhancing local NADPH supply can serve as a reference for improving the flux of compartmentalized synthesis. However, the specific molecular mechanism by which NADPH supply in the mitochondrial compartment affects the cytoplasmic heterologous pathway remains unclear, and the quantitative relationship between the cofactor supply capacity of NADPH-dependent biosynthetic pathways in the mitochondrial compartment and the pathway flux demand has not yet been systematically analyzed.

## 4 Potential developments in mitochondrial compartmentalization

The mitochondrial compartmentalization strategy of *S. cerevisiae* shows irreplaceable application potential in the field of terpenoid synthesis. However, its development is still limited by core issues such as insufficient physical space, the carrying capacity of mitochondria, a lack of a dynamic regulation system, low efficiency of metabolite transmembrane transport, and difficulty in optimizing multivariable systems. In response to the above research gaps, future technological breakthroughs need to focus on the in-depth integration of the biological characteristics of mitochondria themselves and the industrial needs of synthetic biology, and be systematically promoted from the following four interrelated and complementary dimensions.

First, carry out targeted engineering modification of the overall mitochondrial morphology and inner membrane system. Existing studies have confirmed that the volume, quantity, and morphological structure of mitochondria in *S. cerevisiae* can be directionally altered by regulating the expression of fission- and fusion-related genes, and the total mitochondrial volume is significantly positively correlated with the yields of mitochondria-targeted terpenoid precursors and end products, which provides clear experimental evidence for subsequent exploration in this direction (Jia et al., 2020; Yanagibashi et al., 2024; Kichuk and Avalos, 2025). Subsequent work can expand the overall mitochondrial volume to enhance the capacity of the matrix space to accommodate the heterologous mevalonate pathway and terpenoid synthases; meanwhile, explore the effect of inner membrane structure changes on the transmembrane transport efficiency of pyrophosphate intermediates such as IPP/DMAPP, so as to alleviate the cytotoxicity caused by metabolite accumulation. In addition, it is also feasible to explore increasing the number of mitochondria by regulating fission-related genes, and verify the actual effect of strengthening the compartmentalization isolation and reducing the consumption of precursor metabolites by competing pathways in the cytoplasm. Given that existing studies (Yanagibashi et al., 2024) have shown that mitochondrial morphology modification easily triggers strain growth defects, subsequent work needs to focus on coordinating the relationship between morphology optimization, product synthesis, and basic cell growth, weakening the negative impact of morphology modification on normal cell functions, and realizing the synergistic improvement of synthesis capacity and growth homeostasis.

Second, establish efficient metabolite exchange channels between mitochondria, the cytoplasm, and other organelles. The double membrane structure of mitochondria isolates toxic intermediates and optimizes the reaction microenvironment. However, it also restricts the efficient input of precursor substances and the timely output of end products, thus becoming a key bottleneck that limits the improvement of terpenoid synthesis efficiency. On one hand, it is necessary to systematically identify and engineer the endogenous mitochondrial carrier family to significantly enhance its ability to export key pyrophosphate intermediates. On the other hand, the general strategies for transporter discovery and engineering can be used as a reference to reshape the substrate specificity of mitochondrial carriers. Meanwhile, new transport mechanisms such as artificial membrane vesicle transport can be explored to achieve the directional and efficient transport of hydrophobic terpenoid products or highly toxic intermediates (Lv et al., 2022). As a novel substance exchange mechanism independent of traditional transporters, artificial membrane vesicle trafficking system provides a brand-new idea for breaking the metabolite transport bottleneck posed by the mitochondrial membrane. Existing studies have confirmed that by introducing only minimal elements such as core vesicle fusion components and targeting affinity tags, artificial vesicles can be endowed with the ability to specifically recognize and fuse with target organelles, realizing precise intracellular substance delivery (Koike and Jahn, 2017). Compared with protein transporters, membrane vesicles are naturally compatible with the physicochemical properties of hydrophobic terpenoids, and can bypass the energy barrier and substrate specificity restriction in transmembrane transport. At present, the feasibility of vesicle-mediated transmembrane transport of hydrophobic metabolites has been verified in both prokaryotic and eukaryotic systems, and the relevant engineering strategies can provide proof-of-concept support and modular design references for applications in the yeast mitochondrial scenario (Koike and Jahn, 2017; Wu et al., 2019; Reifenrath et al., 2020; Son et al., 2022). In the future, a mitochondria-targeting artificial vesicle transport module can be constructed relying on the endogenous inner membrane synthesis system and microtubule trafficking pathway of *S. cerevisiae*, to unblock the channels for precursor import and product export, and further unlock the performance potential of compartmentalized synthesis.

Third, explore the synergistic system of multienzyme ordered assembly and mitochondrial compartmentalization. Most existing mitochondrial compartmentalization modification strategies achieve only subcellular localization of metabolic pathways, while neglecting the spatial ordered arrangement of multiple catalytic enzymes within the compartment. In recent years, Sun et al. (2022) developed the mimic PKS enzyme assembly line (mPKSeal) strategy, which employs the docking domains of type I *cis*-AT polyketide synthases to recruit the modular assembly and co-localization of cascade metabolic enzymes, significantly improving biocatalytic efficiency and target product production. Based on this, it is speculated that combining such modular docking elements with mitochondrial targeting strategies is expected to construct a dual-optimized system that integrates both compartmentalization isolation and ordered enzyme organization in eukaryotic mitochondria. The orthogonal interaction feature of the docking domains between heterologous enzymes in this strategy theoretically provide new engineering paradigms for the spatial organization modification of the terpenoid biosynthetic pathway in *S. cerevisiae* mitochondria.

Fourth, introduce a rational design method assisted by machine learning to achieve multidimensional global optimization of the mitochondrial compartmentalized synthesis system. The mitochondrial compartmentalized terpenoid synthesis system involves the synergistic action of multiple high-dimensional variables, including targeting signal efficiency, enzyme combination compatibility, cofactor supply balance, and mitochondrial morphological parameters. Traditional trial-and-error optimization methods have inherent drawbacks such as long cycles, high costs, and low efficiency. Existing computational tools can accurately predict mitochondrial targeting efficiency at the whole proteome scale, and machine learning techniques have shown significant advantages in genome-scale metabolic model construction, multi-step synthesis pathway optimization, and gene regulatory element design (Almagro Armenteros et al., 2017; Cheng et al., 2023). Integrating these mature computational frameworks deeply with mitochondrial compartmentalized design can quickly screen the optimal enzyme localization combinations, MTS sequences, and mitochondrial morphological parameters in the virtual space, greatly reducing the trial-and-error costs of wet experiments and promoting a fundamental transformation of terpenoid synthesis in *S. cerevisiae* mitochondria from empirical modification to rational and systematic design.

In conclusion, the synthesis of terpenoids through mitochondrial compartmentalization in *S. cerevisiae* is moving from an experience-driven stage to a new phase of rational design. With the continuous in-depth research on mitochondrial biology and the continuous iteration of synthetic biology tools, it is expected that a mitochondrial synthesis platform with better performance and higher universality can be constructed in the future. This will not only significantly improve the industrial production efficiency of high-value terpenoids but also provide important theoretical references and technical paradigms for the synthetic biology transformation of other eukaryotic organelles.
